# Diagnostic Accuracy of Methods for Detection of Antibodies against Type I Interferons in Patients with Endocrine Disorders

**DOI:** 10.3390/jpm12121948

**Published:** 2022-11-24

**Authors:** Nurana Nuralieva, Marina Yukina, Leila Sozaeva, Maxim Donnikov, Liudmila Kovalenko, Ekaterina Troshina, Elizaveta Orlova, Dmitry Gryadunov, Elena Savvateeva, Ivan Dedov

**Affiliations:** 1 Endocrinology Research Centre, Ministry of Health of Russia, 117036 Moscow, Russia; 2 Medical Institute, Surgut State University, 628416 Surgut, Russia; 3 Center for Precision Genome Editing and Genetic Technologies for Biomedicine, Engelhardt Institute of Molecular Biology, Russian Academy of Sciences, 119991 Moscow, Russia

**Keywords:** autoantibodies, type I interferon, interferon-ω, interferon-α2, multiplex assay, protein microarray, cell-based autoantibody assay, ELISA

## Abstract

Autoantibodies against type 1 interferons (IFN-I) are a highly specific marker for type 1 autoimmune polyglandular syndrome (APS-1). Moreover, determination of antibodies to omega-interferon (IFN-ω) and alpha2-interferon (IFN-α2) allows a short-term diagnosis in patients with isolated and atypical forms of APS-1. In this study, a comparison of three different methods, namely multiplex microarray-based, cell-based and enzyme-linked immunosorbent assays for detection of antibodies against omega-interferon and alpha2-interferon, was carried out. A total of 206 serum samples from adult patients with APS-1, APS-2, isolated autoimmune endocrine pathologies or non-autoimmune endocrine disorders, and healthy individuals were analyzed. In the APS-1 patient cohort (*n* = 18), there was good agreement between the results of anti-IFN-I antibody tests performed by three methods, with 100% specificity and sensitivity for microarray-based assay. Although only the cell-based assay can determine the neutralizing activity of autoantibodies, the microarray-based assay can serve as a highly specific and sensitive screening test to identify anti-IFN-I antibody positive patients.

## 1. Introduction

Autoantibodies against type 1 interferons (IFN-I) are highly specific for type 1 autoimmune polyglandular syndrome (APS-1), a monogenic disease caused by a mutation in the *AIRE* gene [1]. The formation of these autoantibodies (auto-Abs) is presumably caused by a disorder in the negative selection for IFN-I-specific T-lymphocytes in the thymus [2]. For APS-1 patients, it has been shown that Abs against omega-interferon (IFN-ω) are 100% specific and antibodies against alpha2-interferon (IFN-α2) are 99.9% specific [3]. However, such auto-Abs are also typical for patients with myasthenia gravis and/or thymoma [4], and can be detected in patients with systemic lupus erythematosus, rheumatoid arthritis [5], Sjogren’s syndrome [6], and incontinentia pigmenti [7].

Nevertheless, recent data indicate that anti-interferon auto-Abs are more common in the population than previously thought. Over the past two years, the role of auto-Abs against IFN-I in SARS-CoV-2 infection has been demonstrated [7]; moreover, they have been shown to underlie severe side effects after vaccination with a live attenuated yellow fever virus vaccine [8]. Multiple studies have shown that more than 10% of patients with neutralizing auto-Abs against IFN-I had life-threatening COVID-19 pneumonia [9,10,11]. The presence of auto-Abs against IFN-I appears to remain clinically asymptomatic in people prior to their infection with SARS-CoV-2. To date, a case of APS-1 was diagnosed in two brothers, 7-year-old and 13-year-old, with life-threatening pneumonia caused by COVID-19. Due to the severity of COVID-19 and their medical history, APS-1 was suspected and the patients underwent appropriate molecular testing. In addition to a mutation in the *AIRE* gene, the patients were found to carry auto-Abs against IFN-I [12]. Thus, the long-standing opinion that there is no increased susceptibility to viral infections in patients with APS-1, despite the presence of auto-Abs against IFN-I [13], is refuted. Currently, there are also suggestions about the possible role of autoantibodies against IFN-I in other severe viral and malignant diseases, especially in the elderly [14].

At the same time, the work on the development and implementation of screening tests for anti-cytokine antibodies, including antibodies against IFN-1 for patients with endocrine diseases, is in progress. For instance, recently, Sjøgren et al., using screening tests for auto-Abs against IFN-ω and interleukin-22 (IL-22) on a large cohort of patients with endocrine diseases, followed by subsequent genetic testing of positive samples, identified patients with undiagnosed APS-1 as well as several patients with previously unknown monogenic or oligogenic causes of organ-specific autoimmunity and immunodeficiency [15]. Identification of patients with endocrine autoimmune conditions is important to ensure targeted treatment and personalized follow-up aimed at preventing complications. Thus, the development of new effective methods for the detection of auto-Abs against IFN-I is extremely important. The simplicity of the assay and its availability is a priority for conducting exploratory research and screening tests.

There are various methods for detecting auto-Abs against IFN-I, for example, antiviral interferon neutralizing assay [1], time-resolved immunofluorometric assay [2], radioimmunoassay [16], magnetic-beads-based assay [17] and microarray-based assay [18], as well as some commercially available enzyme-linked immunosorbent assays (ELISA). Until now, in Russia, the study of auto-Abs against IFN-I was carried out for the purpose of APS-1 diagnostics using a cell-based autoantibody assay (CBAA) [19]. Orlova et al., who introduced the method for the qualitative determination of auto-Abs against IFN-ω and IFN-α using the human embryonic kidney (HEK)-blue cell culture in Russia, confirmed its high sensitivity and specificity for APS-1 [20,21]. However, due to the need for cell cultivation, this method is rather laborious.

Earlier, our research group developed and tested a microarray to detect auto-Abs associated with endocrine autoimmune diseases, both organ-specific (auto-Abs against thyroperoxidase (TPO), thyroglobulin (Tg), glutamic acid decarboxylase (GAD-65), islet cell cytoplasmic antigen (ICA), tyrosine phosphatase-like protein (IA2) and steroid 21-hydroxylase (21-OH)) and anti-cytokine autoantibodies (anti-IFN-ω, anti-IFN-α-2a and anti-interleukin 22(IL-22)-auto-Abs) [22]. Using this microarray, we detected a characteristic triplet of anti-cytokine auto-Abs in 89% of patients with APS-1 in the studied cohort; 100% of them were found to carry auto-Abs against IFN-I. However, a method to compare auto-Abs against IFN-I was not available in this study. The aim of this work was to compare the specificity and sensitivity of anti-IFN-I auto-Abs detection using various methods: multiplex microarray analysis, CBAA and a commercially available ELISA.

## 2. Materials and Methods

### 2.1. Patients, Healthy Donors and Serum Samples

The study included 206 participants: the main group—18 patients with APS-1 (group 1), and three control groups—89 patients with autoimmune endocrine pathology (group 2), 71 patients with non-autoimmune endocrine pathology (group 3) and 28 healthy individuals (group 4).

Group 2 included patients with the following pathologies:

- Autoimmune polyendocrine syndrome type 2, *n* = 38;
- Autoimmune thyropathies (autoimmune thyroiditis (AIT) and Graves’ disease), *n* = 23;
- Type 1 diabetes mellitus (T1D)/latent autoimmune diabetes in adults (LADA), *n* = 21;
- Hypergonadotropic hypogonadism (HH) of autoimmune origin, *n* = 4;
- Autoimmune adrenal insufficiency (AAI), *n* = 3.

Group 3 included patients with the following pathologies:

- Non-autoimmune thyroid diseases, *n* = 5;
- Non-autoimmune diabetes, *n* = 5;
- Non-autoimmune HH, *n* = 6;
- Non-autoimmune adrenal insufficiency (AI), *n* = 17;
- Non-autoimmune pathology of the parathyroid glands, *n* = 6;
- Multiple endocrine pathology of non-autoimmune origin, *n* = 32.

Group 4 included healthy individuals, in particular, carriers of antibodies that are markers of autoimmune damage of the pancreatic islet apparatus without carbohydrate metabolism disorders. In addition, group 4 included the parents of a patient with APS-1 (#194 in Table S1) who were heterozygous carriers of a mutation in the *AIRE* gene without any diagnosed autoimmune diseases.

Criteria for inclusion and exclusion from this study are summarized in Table S2. The characteristics of the participants are provided in Tables S1 and S3. Before inclusion into the study, a comprehensive examination of all patients and healthy individuals was carried out. To clarify the genesis of the disease, all participants underwent an examination of the level of organ-specific autoantibodies using the following assays: ELISA (anti-21-OH (BioVendor, Czech Republic), anti-GAD (Euroimmun, Germany), anti-IA2 (Medipan Gmbh, Berlin, Germany), anti-ICA (Medipan Gmbh, Berlin, Germany)) and chemiluminescent cicroparticle immunoassay (CMIA) (anti-TPO (Abbott Laboratories, Chicago, IL, USA), anti-TG (Roche Diagnostics, Switzerland)) (Table S1).

Patient serum samples were frozen and stored at −80 °C until analysis.

### 2.2. Microarray-Based Assay

Hydrogel microarrays with immobilized antigens, including IFN-ω (Cat # 300-02J, PeproTech, Cranbury, NJ, USA) and IFN-α-2a (Cat # 11100-1, PBL Assay Science, Piscataway, NJ, USA), were manufactured as described previously [22]. Each antigen was immobilized in 4 repetitions to increase the reproducibility of the assay results. Patient serum samples were diluted 1:100 (100 mM Tris-HCl buffer with 0.1% Triton X-100, Sigma-Aldrich, St. Louis, MO, USA) and applied to the microarray (100 µL). After overnight incubation at 37 °C, intermediate washing for 20 min (PBS with 0.1% Tween 20, Sigma-Aldrich, St. Louis, MO, USA), rinsing and drying, the microarrays were developed with fluorescently labeled anti-species antibodies. As detecting antibodies, we used F(ab’)2-Goat anti-Human IgG Fc gamma Secondary Antibody (Cat # 31163, Invitrogen, Waltham, MA, USA) labeled with Cy5 cyanine dye. After incubation for 30 min at 37 °C, the microarrays were washed (PBS with 0.1% Tween 20, 30 min), rinsed and dried by centrifugation. Registration of fluorescent images of microarrays and calculation of fluorescent signals were performed using a microarray analyzer and software developed at the Engelhardt Institute of Molecular Biology, Moscow. Interpretation of the results of analysis on a microarray with the determination of the presence/absence of auto-Abs against IFN-ω and IFN-α-2a in blood serum were performed as described previously [22]. The determination of anti-IFN-ω and anti-IFN-α-2a by the microarray-based multiplex analysis was carried out in serum samples from all participants in the study (Table S1).

### 2.3. Anti-IFN-ω Cell-Based Autoantibody Assay

The study of auto-Abs against IFN-ω was carried out by the method of qualitative determination using the HEK-blue cell culture according to a previously developed method [19]. The method is based on the ability of cells to synthesize alkaline phosphatase in the presence of IFN. With a high titer of neutralizing IFN antibodies in the patient’s serum, the ability of the cell to synthesize alkaline phosphatase is suppressed, which in turn is registered by measuring the optical density. The presence of secreted alkaline phosphatase is determined using a spectrometer with the determination of the optical density of the test liquid at a wavelength of 650 nm (Abs650). IFN inhibition is given as a percentage of neutralization (Abs index) and is calculated by the formula:

$$\mathrm{Abs}\;\mathrm{index}=\frac{\mathrm{Abs}650\;\mathrm{negative}\;\mathrm{control}-\mathrm{Abs}650\;\mathrm{sample}}{\mathrm{Abs}650\;\mathrm{negative}\;\mathrm{control}-\mathrm{Abs}650\;\mathrm{positive}\;\mathrm{control}}$$

Determination of auto-Abs against IFN-ω using the HEK-blue cell culture was carried out in 200 study participants: group 1–17 patients with APS-1, and control groups (groups 2–4)—87 patients with autoimmune endocrine pathology, 68 patients with non-autoimmune endocrine pathology and 28 healthy individuals, respectively (Table S1).

### 2.4. Anti-IFN-α Autoantibodies ELISA

To detect autoantibodies against IFN alpha, serum samples were tested by ELISA according to the manufacturer’s instructions (Cat # BMS217, Invitrogen, Waltham, MA, USA). The determination of auto-Abs against IFN-α by ELISA was carried out for positive samples identified using a microarray-based assay, and selectively for 10 negative samples. In total, the determination of auto-Abs against IFN-α by ELISA was carried out for 29 study participants: 17 patients with APS-1 (group 1), 2 patients with non-autoimmune endocrine pathology (group 3) and 10 healthy individuals (group 4) (Table S1).

## 3. Results and Discussion

### 3.1. Autoantibodies against IFN-I among APS-1 Patients

In this work, a comparison of three different methods, namely multiplex microarray-based, cell-based and enzyme-linked immunosorbent assays for anti-IFN-I antibody detection, was carried out on a sample of APS-1 patients, patients with autoimmune endocrine pathologies and non-autoimmune endocrine pathologies, and healthy individuals. The results of detection of autoantibodies against IFN-I using microarray-based assay, CBBA and ELISA for APS-1 patients are summarized in Table 1.

**Figure 1:**
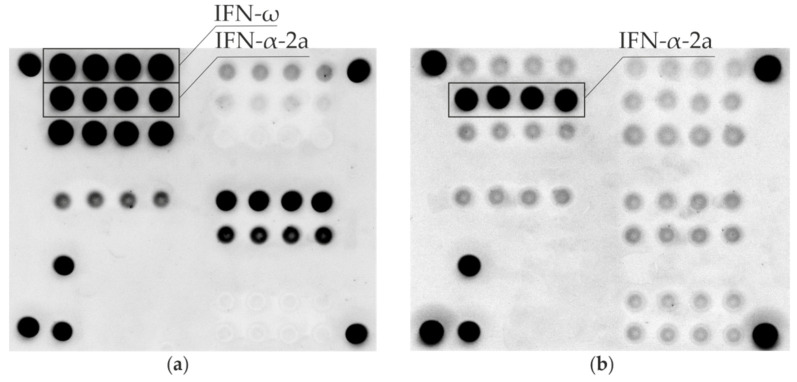
Fluorescence images of microarrays after assay with serum samples from: an APS-1 patient (**a**), a patient with multiple endocrine neoplasia type 1 (**b**). Groups of elements with immobilized omega-interferon (IFN-ω) and alpha2a-interferon (IFN-α-2a) are indicated by solid lines.

Samples from 206 patients were analyzed using a previously developed microarray for multiplex detection of auto-Abs [22]. All APS-1 patients (*n* = 18) were positive for auto-Abs against IFN-ω and IFN-α-2a. As an example, Figure 1a shows a fluorescent image of a microarray after assay with an APS-1 patient sample. According to the previously described algorithm for interpreting the results [22], positive signals indicating the presence of auto-Abs in blood serum were detected for groups of elements containing IFN-ω and IFN-α-2a, as well as for groups of elements containing IL-22, 21-OH, GAD-65, TG and TPO (not considered in this work). Figure 1b shows the results of microarray-based assay with serum from a patient with multiple endocrine neoplasia type 1. Positive signals indicating the presence of autoantibodies in serum were detected only for a group of elements containing IFN-α-2a.

Using the CBAA method [19], samples from 200 patients were analyzed for auto-Abs against IFN-ω. All analyzed APS-1 samples (*n* = 17), except for one, were anti–IFN–ω auto-Abs positive. A negative anti-IFN-ω auto-Abs result was detected using CBAA in APS-1 patient #135 with a common *AIRE* mutation and the classic triad (Table 1). For technical reasons, it was not possible to re-examine this sample by CBAA. On the one hand, the results are consistent with data of other researchers: for example, the absence of auto-Abs against IFN-ω in patients with mutations in *AIRE* and only one component of APS-1 (hypoparathyroidism) was observed in the work of Cervato et al. [23]. On the other hand, patient #135 was shown by microarray-based multiplex assay to carry both auto-Abs against IFN-ω and auto-Abs against IFN-α; the presence of the latter was also confirmed by ELISA.

Using a commercially available ELISA, samples from 29 patients (among them, 17 APS-1 patients) were analyzed for auto-Abs against IFN-α. For anti-IFN-α auto-Abs, an agreement between the results of the two methods (microarray-based assay and ELISA) was observed; all analyzed APS-1 patient samples were anti-IFN-α auto-Abs positive for both methods (Table 1). Since the ELISA kit was quantitative, it is worth noting that all positive samples were oversaturated (˃1 µg/mL) on initial analysis. For individual samples, the concentration was estimated at a 5000-fold dilution or more, resulting in a concentration of anti-IFN-α antibodies in the samples, on average, of about 1 mg/mL, taking into account the dilution.

### 3.2. Anti-IFN-I Autoantibodies among Control Groups

The microarray-based assay identified two patients who were anti-IFN-ω and/or anti-IFN-α-2a auto-Abs positive among patients from groups without diagnosed APS-1. Sample #56 (male, 70 years old) was positive for both anti-IFN-ω and anti-IFN-α-2a auto-Abs, and sample #147 (female, 45 years old) was positive for auto-Abs against IFN-α-2a. These two serum samples were also shown to be anti-IFN-α auto-Abs positive by ELISA. However, serum sample #56 was found to be anti-IFN-ω auto-Abs negative using CBAA. The frequency of detection of anti-IFN-I auto-Abs in the groups by different methods is summarized in Table 2.

Anti-IFN-I auto-Abs positive patients from control group 3 had severe and complex medical conditions. For instance, patient #56, who was tested positive for anti-IFN-ω auto-Abs using the microarray-based assay, was diagnosed with adrenal insufficiency of non-autoimmune origin as a result of adrenal metastasis (presumably, the primary tumor was lymphoma). Using multiplex analysis, auto-Abs against IFN-α2 were also detected in the patient, later confirmed by ELISA. The patient denied that he underwent therapy with IFN drugs, as well as the presence of a thymoma in anamnesis, which may be the cause of the formation of anti-IFN-I auto-Abs [4,24]. During an ophthalmological examination, the presence of myasthenia gravis was not detected.

Anti-IFN-α auto-Abs positive patient #147 (by microarray-based assay and by ELISA) had a genetically proven syndrome of multiple endocrine neoplasia type 1 (multiple insulinomas, non-functioning tumors of the pancreas, duodenal gastrinomas, primary hyperparathyroidism, lung carcinoids, multifocal hormonally inactive formations in both adrenal glands, hyperprolactinemia). The formation of anti-IFN-α auto-Abs in this patient can also be related to antiviral therapy for hepatitis C 17 years prior to participation in this study; similar cases have been described previously [25]. In light of an emerging assumption about a possible role for anti-IFN-I auto-Abs in severe viral or malignant diseases [14], these anti-IFN-I auto-Abs positive patients identified by microarray-based assay are of great interest.

APS-1 is an orphan disease. The prevalence of APS-1 in different European countries averages from 1:90,000 to 1:200,000 [26]. Unfortunately, there are currently no data on the frequency of occurrence of APS-1 in the population in Russia. The largest Russian APS-1 cohort described to date included 112 patients [27]. Since the symptoms may debut at intervals of several years and non-specific clinical manifestations can occur, diagnosis is often delayed. Autoantibodies against IFN-ω and IFN-α2 are specific and sensitive markers of APS-1, which makes it possible to use them for screening diagnostics of patients with autoimmune diseases to exclude atypical forms of the disease. Although there are a number of research tests for the detection of antibodies against IFN-I, including those that allow the determination of the neutralizing activity of autoantibodies, studies aimed at comparing the specificity and sensitivity of these methods in the diagnostics of APS-1 are extremely limited. The main problem of such studies is the small sample of APS-1 patients, which makes it difficult to validate the results. In the present study on a small cohort of APS-1 patients, there was a good agreement between results of the tests for anti-IFN-1 antibodies performed by microarray-based assay and previously validated cell-based autoantibody and enzyme-linked immunosorbent assays. The results indicated that microarray-based assay can serve as a highly specific and sensitive screening test to identify anti-IFN-1 antibody positive patients. The introduction of such tests into clinical practice would make it possible to increase the availability of testing for antibodies against IFN-I in diagnostically difficult cases, as well as to study larger cohorts of patients with various severe viral and malignant diseases.

## 4. Conclusions

Microarray-based assay showed almost 100% sensitivity and specificity in detection of anti-IFN-I antibodies in patients with endocrine disorders. Determination of antibodies to IFN-ω and IFN-α2 can be used as an inexpensive and rapid way to diagnose APS-1. Thanks to these antibodies, the number of people who need to undergo genetic analysis can be reduced, i.e., in patients who do not have antibodies to IFN-ω and IFN-α2, the diagnosis of APS-1 can be ruled out without testing for the *AIRE* gene. In addition, this analysis may be recommended for individuals who do not fully meet the diagnostic criteria: patients who failed to detect a second mutation in the *AIRE* gene, or patients with dominant-negative mutations.

## Figures and Tables

**Table 1:** Characteristics of the type I autoimmune polyglandular syndrome (APS-1) patients and results of testing serum samples for the presence of anti-omega-interferon (IFN-ω) and anti-alpha-interferon (IFN-α) autoantibodies using microarray-based, cell-based and enzyme-linked immunosorbent immunoassays.

Sample #	Age	Gender	*AIRE* Mutations	APS-1 Components							Anti-IFN-ω Auto-Abs		Anti-IFN-α Auto-Abs	
				Endocrine					Non-Endocrine					
				AAI *	Hypoparathyroidism	TD1/LADA	AIT	HH	CMC	Other pathology	Microarray	CBAA	Microarray	ELISA
3	49	m	R257X/R257X	+	+	−	-	-	+	−	+	+	+	*n/a*
49	20	f	R257X/R257X	+	+	−	+	+	+	Malabsorption syndrome, tooth enamel hypoplasia, total alopecia, vitiligo	+	+	+	+
51	18	f	R257X/-	+	+	−	−	+	+	−	+	+	+	+
64	29	f	p.R257 */p.W78R	+	+	−	−	+	+	Atrophic gastritis, cataract	+	+	+	+
103	18	f	R257X/R257X	+	+	−	−	+	+	Atrophic gastritis, cataract	+	+	+	+
115	30	f	R257X/R257X	+	+	+	+	−	+	Total alopecia, atrophic gastritis	+	+	+	+
124	18	m	R257X/A58V	+	−	−	−	−	+	B12 deficiency anemia, malabsorption syndrome, tooth enamel hypoplasia, spleen hypoplasia	+	+	+	+
125	45	f	not studied	+	+	-	+	−	+	Atrophic gastroduodenitis, vitiligo, cataract, corneal dystrophy	+	+	+	+
129	45	m	not studied	+	+	−	−	−	+	Alopecia areata	+	+	+	+
133	27	m	−	+	+	−	+	−	+	Autoimmune fibrosing alveolitis, total alopecia	+	*n/a*	+	+
135	30	f	R257X/R257X	+	+	−	−	+	+	Vitiligo, malabsorption syndrome, corneal dystrophy, partial eyelid ptosis, asplenia, atrophic gastroduodenitis, autoimmune hepatitis	+	*−*	+	+
136	28	f	R257X/c.931delT	+	+	−	−	+	+	Corneal dystrophy, atrophic gastritis	+	+	+	+
152	27	f	R257X/-	+	+	−	−	+	+	Subtotal alopecia, tooth enamel dysplasia, chronic tubulointerstitial nephritis	+	+	+	+
156	36	m	not studied	+	+	−	−	−	+	−	+	+	+	+
168	32	f	R257X/R257X	+	+	−	−	+	+	Diffuse alopecia, vitiligo	+	+	+	+
189	31	f	R257X/R257X	+	+	−	−	+	+	Atrophic gastroduodenitis, malabsorption syndrome, tooth enamel hypoplasia, retinitis pigmentosa, cataract, strabismus	+	+	+	+
191	44	f	R257X/R257X	+	+	−	−	+	+	Cataract	+	+	+	+
194	25	f	R257X/c.821delG	+	+	−	+	+	+	Atrophic gastritis	+	+	+	+

* Abbreviations: m—male; f—female; AAI—autoimmune adrenal insufficiency; T1D/LADA—type 1 diabetes mellitus/latent autoimmune diabetes in adults; AIT—autoimmune thyroiditis; HH—hypergonadotropic hypogonadism; CMC—chronic mucocutaneous candidiasis; CBAA—cell-based autoantibody assay; ELISA—enzyme-linked immunosorbent assay.

**Table 2:** Anti-IFN-I positive patients by group (n/n total), sensitivity and specificity of assays.

Group	Anti-IFN-ω Auto-Abs Microarray	Anti-IFN-ω Auto-Abs CBAA	Anti-IFN-α Auto-Abs Microarray	Anti-IFN-α Auto-Abs ELISA
1	18/18	16/17	18/18	17/17
2	0/89	0/87	0/89	n/a
3	1/71	0/68	2/71	2/2
4	0/28	0/28	0/28	0/10
Total number of samples tested	206	200	206	29
Sensitivity, 95% CI	100.0% [78.9%; 100.0%]	94.1% [70.7%; 100.0%]	100.0% [78.9%; 100.0%]	Not determined. The assay was used as a comparative test
Specificity, 95% CI	99.5% [96.7%; 100.0%]	100.0% [97.5%; 100.0%]	98.9% [95.9%; 99.9%]	Not determined. The assay was used as a comparative test

## Data Availability

The authors confirm that the data supporting the findings of this study are available within the article and/or its Supplementary Materials.

## Supplementary Materials

The following supporting information can be downloaded at: https://www.mdpi.com/article/10.3390/jpm12121948/s1, Table S1: Patient characteristics and results of testing serum samples for the presence of autoantibodies using microarray-based assay, CBAA, ELISA and CMIA. Table S2: Criteria for inclusion and exclusion from this study; Table S3: Age and gender in groups of participants.
