# Supplementary material for: Diagnostic Accuracy of Methods for Detection of Antibodies against Type I Interferons in Patients with Endocrine Disorders
Source: J Pers Med. 2022 Nov 24;12(12):1948. doi: 10.3390/jpm12121948 (PMC9783777; doi:10.3390/jpm12121948)
Supplement: Supplementary file 1 [file jpm-12-01948-s001.zip › Table S2. Criteria for inclusion and exclusion of patients in the study.pdf]

Table S2. Criteria for inclusion and exclusion of patients in the study

**Common criteria for inclusion in groups 1-4:**
Male and female.<br>Age: 18 and older.<br>Signed informed consent, approved by the local ethics committee of the Endocrinology Research Centre, Ministry of Health of Russia, Moscow, Russia (protocol .17 and date of approval 27 September 2017).
**Additional criteria for inclusion in group 1:**
At least two major components of APS-1 and/or mutation in the *AIRE* gene.
**Additional criteria for inclusion in group 2:**
Autoimmune thyroiditis: Elevated levels of antibodies to TPO, TG and/or TSHR; signs of autoimmune lesion on thyroid ultrasound and/or medical history (in the case of Graves' disease).
Autoimmune diabetes mellitus: the onset of the disease from a young age, and/or an increased level of antibodies to GAD-65, ZnT8, IA2, IAA and/or ICA.
Hypergonadotropic hypogonadism of autoimmune genesis: the acquired form is excluded.
Primary autoimmune adrenal insufficiency: the acquired form is excluded.
**Additional criteria for inclusion in group 3:**
No criteria for inclusion in groups 1-3 (according to the survey).
Non-autoimmune thyroid disease: normal level of AT to TPO, TG, and/or TSHR; no signs of autoimmune damage according to ultrasound of the thyroid gland; intact thyroid gland; lack of concomitant autoimmune diseases.
Non-autoimmune diabetes mellitus: normal levels of antibodies to GAD-65, ZnT8, IA2, IAA, and/or ICA; intact pancreas; lack of concomitant autoimmune diseases.
Non-autoimmune hypergonadotropic hypogonadism: intact ovaries, testes; lack of concomitant autoimmune diseases.
Non-autoimmune adrenal insufficiency: intact adrenal glands, normal level of AT to 21-OH; lack of concomitant autoimmune diseases.
Non-autoimmune parathyroid disease: absence of concomitant autoimmune diseases and intact parathyroid gland.
**Additional criteria for inclusion in group 4:**
Absence of endocrine (autoimmune and non-autoimmune) pathology (according to the survey).
**Criteria for exclusion (all groups):**
Pregnancy, lactation.<br>Acute infections.<br>Severe, life-threatening conditions: decompensation of chronic heart failure, chronic kidney disease C3b or more, pulmonary and liver failure, mental illness.<br>Other immune system pathology (including congenital and acquired immunodeficiencies, hypersensitivity reactions).<br>Taking drugs that affect immune system function (interleukins, interferons, immunoglobulins, immunosuppressants, cytostatics) for 6 weeks before inclusion in the study. Vaccinations/revaccinations within a month before inclusion in the study.
