# Supplementary material for: Diagnostic Accuracy of Methods for Detection of Antibodies against Type I Interferons in Patients with Endocrine Disorders
Source: J Pers Med. 2022 Nov 24;12(12):1948. doi: 10.3390/jpm12121948 (PMC9783777; doi:10.3390/jpm12121948)
Supplement: Supplementary file 1 [file jpm-12-01948-s001.zip › Table S3. Age and gender in groups of participants.pdf]

Table S3. Age and gender in groups of participants

Group	Patients				
	n	Age (years)	Gender (Femail/Male)		
			n	%	ratio
1	18	18-49	13/5	72/28	2,6:1
2	89	19-72	75/14	84/16	5,4:1
3	71	18-88	51/20	72/28	2,6:1
4	28	18-60	22/6	79/21	3,6:1
